# Preparation of modified shiitake mushroom stem insoluble dietary fiber and black rice anthocyanin complex and its effects on the gel properties, rheological properties and microstructure of rice starch

**DOI:** 10.1016/j.fochx.2026.104344

**Published:** 2026-08-20

**Authors:** Hongxiu Fan, He Gao, Yumeng Zhang, Meixuan Du, Tingting Liu, Dawei Wang, Hongcheng Liu, Yanrong Zhang

**Affiliations:** a School of Food Science and Engineering, Jilin Agricultural University, Changchun 130118, China; b Scientific Research Base of Edible Mushroom Processing Technology Integration of Ministry of Agriculture and Rural Affairs, Changchun 130118, China; c Engineering Research Center of Grain Deep-processing and High-efficiency Utilization of Jilin Province, Changchun 130118, China; d Key Laboratory of Technological Innovations for Grain Deep-processing and High-efficiency Utilization of By-products of Jilin Province, Changchun 130118, China

**Keywords:** Insoluble dietary fiber-anthocyanin complex, Shiitake mushroom stem, Interaction mechanism, Physicochemical property

## Abstract

This study constructed a complex of steam-exploded shiitake mushroom stem insoluble dietary fiber (SEIDF) and black rice anthocyanins (BRA) and elucidated the interactions between SEIDF and BRA, the structural features of the SEIDF-BRA complex, and the effect of this complex on the rice starch gel structure and property. Results showed that SEIDF had higher surface porosity and roughness after steam explosion. BRA was adsorbed onto SEIDF in a multilayer manner, and the interaction between BRA and SEIDF involved non-covalent binding (including hydrogen bonds), resulting in the formation of a thermally stable complex. Structure characterization indicated that the hydrogen bonding of the SEIDF-BRA complex was greatly enhanced, and more functional groups (such as C—O) were exposed in the SEIDF-BRA complex. Moreover, the addition of the SEIDF-BRA complex not only increased the gelatinization temperature and viscosity of rice starch while inhibiting starch retrogradation, but also improved the rheological behavior, texture property and sensory quality of rice starch. Structurally, the SEIDF-BRA complex reduced the relative crystallinity and short-range order degree of rice starch and improved the porosity and cross-linking of the gel network. This study might contribute to the development of SEIDF-BRA complex as a potential functional food ingredient.

## Highlights

• SEIDF and BRA interacted through hydrogen bonds to form a stable complex.
• SEIDF improved thermal stability of BRA under high-temperature conditions.
• RS gel network was optimized by SEIDF-BRA complex mainly via hydrogen bonds.
• SEIDF-BRA complex is a promising functional ingredient for healthier starchy foods.

## 1 Introduction

Rice serves as a fundamental staple food across numerous regions, nourishing more than half of the global population (Gan et al., 2023). Rice starch (RS) accounts for 75% to 80% of the rice composition and is one of the main ingredients in foods, functioning as a calorie provider, texture modifier and low-calorie fat replacement (Li et al., 2024). However, starch-based foods generally lead to a rapid increase in post prandial blood sugar levels. Long-term or excessive consumption may lead to health risks including diabetes. In recent year, consumers have paid more and more attention to the health and nutrition of food, especially functional foods containing natural ingredients. Therefore, the development of functional food with exogenous nutritional supplements has become a research hotspot.

Anthocyanins are a kind of water-soluble polyphenol that are widely distributed in dark plants such as black rice, purple sweet potatoes, blueberries, mulberries, etc. They have various physiological activities, including antioxidants, tumor-inhibiting, lipid-lowering, and blood sugar-lowering effects. Due to these biological functions, anthocyanins have gained increasing attention as nutraceutical and functional food ingredients. They have been added into various products such as noodles, cakes, steam bread and beverages. They not only impart attractive colors to food but also promote health. Pelargonidin, for instance, extracted from strawberries and cranberries, exhibits a vivid red and can serves as a natural coloring agent in beverages and fruit juices. Cyanidin, derived from blackberry and grape skins, displays a purplish-blue color and is suitable for use in beverages and staple foods (such as steam breads and cakes) (Li et al., 2021; Saudar et al., 2026). These two anthocyanins possess potent antioxidant properties and have demonstrated anti-inflammatory, neuroprotective and cardiovascular protective activities. Therefore, anthocyanins have great potential as natural pigment and functional agents in staple food. To transform the potential of anthocyanins into practical applications in starch-based food, selecting a source that balances high bioactive content, abundant sources and economic viability is crucial. Black rice anthocyanin (BRA), which is abundant in black rice bran or black rice skin layer, meets these criteria. The BRA content in black rice bran is extremely high, with some varieties reaching over 300 mg per 100 g (Chen et al., 2024). Owing to their bright colors and health promoting properties including potent antioxidant, anti-inflammatory, anti-atherosclerosis, anti-cancer and anti-obesity effects, BRA have emerged as promising functional ingredients (Huang et al., 2025). However, anthocyanins have low stability under diverse environmental conditions, which limit their practical application. Thus, improving the stability of BRA through absorbing or entrapping by natural macromolecules has elicited considerable attention in functional food development.

Dietary fiber (DF), recognized as the “seventh essential nutrient”, plays an indispensable role in promoting human health. Its functional properties include regulating blood lipids and blood sugar, promoting digestive health, preventing constipation, obesity and chronic diseases. DF can be classified into soluble (SDF) and insoluble (IDF) types based on solubility (Zhao et al., 2025). Increasing evidence has shown that polyphenols can bind to DF spontaneously to form stable conjugates. Since DF is characterized by rough surface topography and porous microstructure, this enables polyphenolic compounds to combine with DF through either entrapment or adsorption mechanisms. Moreover, the hydrophilic hydroxyl groups and hydrophobic aromatic rings of polyphenols enabled them to interact with DF at multiple sites on the surface of plant cell walls via hydrophobic interactions, hydrogen bonds and covalent bonds. Thus, the concept of “antioxidant dietary fiber” has been proposed, which have gained significant interest in functional foods and nutraceutical development as this polyphenolic-DF conjugate combines the physiological properties of both polyphenols and DF. Previous studied have published on the positive effect of polyphenols combined with SDF. Lei et al. (2020) prepared complexes with lotus root SDF and two polyphenols by isothermal adsorption equilibrium method. They reported that the complex presented loose and porous appearance of microstructure and increased thermal stability. Hu et al. (2023) build several fenugreek SDF-polyphenols complexes based on these natural interactions between them. They found that the interaction of fenugreek SDF with desmethoxycurcumin was stronger than that with curcumin, and the antioxidant capacity and solubility of these polyphenols in the fenugreek SDF-polyphenols complexes was enhanced, which was a preliminary in vitro demonstration of SDF to improve the bioavailability of polyphenols. Liu et al. (2024) confirmed that the lotus root SDF-polyphenols complex had higher oil adsorption capacity than the lotus root SDF. Ji et al. (2020) showed that a complex of dietary fiber and resveratrol exhibited higher solubility and stability compared with resveratrol. In addition, the interactions between bound polyphenols and DF, and the effects of bound polyphenols in DF on health problems have been investigated in previous studies. Polyphenols could form interactions with SDF via covalent (such as ester) or non-covalent bonds (such as hydrogen bonding and electrostatic) interactions (Bermúdez-Oria et al., 2019), and the interactions between polysaccharides and phenolic compounds can enhance their beneficial properties, such as ensuring prolonged circulation of released phenolic metabolites and enhancing free radical scavenging abilities. The bound polyphenols in DF exhibited anti-inflammatory, antitumor and neuroprotective activities. All the above studies indicated the polyphenol-DF complex could be used as a functional food ingredient that could potentially promote health. However, there are still some aspects not fully elucidated, including the practical application of the polyphenol-DF complex in health-oriented food products (such as starch-based products), how the polyphenol-DF complex influences the product quality and shelf life, the effect of various environment variables of food processing (such as presence of starch, protein or fats) on the specific property and structure changes of the polyphenol-DF complex. In addition, as different DF possesses different structure and physicochemical properties, the interactions between DF and polyphenols are complex, which could affect the functional, structural and nutritional properties of the complexes. Thus, more details about the mode of interactions of polyphenols with various sources of DF need to be studied to boost the fully utilization of DF and polyphenols.

Shiitake mushrooms are among the most widely cultivated and consumed mushrooms in the world. Their stem is a major by-product in shiitake mushroom processing, accounting for approximately 25–30% of the total mass. The content of IDF in the shiitake mushroom was more than 60–70% (Liu et al., 2019). The high IDF content of shiitake mushroom stems indicates they can serve as a quality source of IDF extraction. However, due to their fibrous texture, shiitake mushrooms stems are difficult to chew, swallow and digest, which lead to their frequent disposal during the harvesting and food processing stages. According to a survey conducted by the China Edible Fungi Association, the annual production of shiitake mushrooms in China reached 13.13 million tons in 2022, with the annual production of mushroom stems estimated to be approximately 3.28 to 3.94 million tons. Discarding these stems as waste would lead to rotting and environmental contamination. Thus, the high-value added utilization of byproducts could eliminate about 3 million tons of mushroom stem waste per year in China. Moreover, shiitake mushroom stems are rich in various nutrients including dietary fiber, polysaccharides, protein and ergosterol, which positions them as a promising resource for the development of functional ingredient (such dietary fiber powder) and heal promoting food products (such as vegetarian meat jerky and meat floss). For instance, utilizing 1 million tons of shiitake mushroom stem as material for dietary fiber powder is projected to generate an output value of 5 billion dollars. Thus, development of shiitake mushroom stems can not only reduce the waste of resources, save costs but also improve economic benefits.

However, the structure of natural DF is typically compact, which inhibit the exposure of its functional groups and lead to poor adsorption capacity. Steam explosion has been extensively utilized in the physical modification of DF and has the advantage of being environmentally friendly, efficient and free of chemical residuals. Specifically, steam explosion cleaves the surface of DF to expose more functional groups, thereby improving the adsorption capacity for cholesterol, bile acids and glucose (Ma et al., 2025). Tian et al. (2024) reported that steam explosion treatment improved the structure and functional characteristics of rice bran. The relative crystallinity and polymerization degree of crystalline regions in rice bran IDF was decreased, the thermal stability was increased, and functional properties including water-holding and oil-holding capacity, glucose adsorption capacity, *α*-amylase and pancreatic lipase inhibition capacity of rice bran IDF were improved. Our previous studies also proved that the complex of the steam exploded shiitake mushroom stem DF and tea polyphenol had higher thermal stability and stronger adsorption capacity for fats, cholesterol and cholates (Chen, Cai, et al., 2025; Chen, Liu, et al., 2025; Chen, Wang, et al., 2025). Thus steam explosion treatment will be utilized in this study to modify shiitake mushrooms steam IDF to improve its functional properties and adsorption capability for polyphenol.

To our best knowledge, most studies focus on the interactions between SDF and exogenous polyphenols and the properties of the SDF-polyphenol complexes, but research on the IDF and polyphenol complexes is limited. In addition, systematic investigations examining how the DF-polyphenol complex influences the property of rice starch, particularly from the perspective of reorganized starch multi-scale structure remain insufficient. Therefore, in this study, a complex of steam-exploded shiitake mushroom stem IDF (SEIDF) and BRA was prepared based on adsorption mechanism, and the effect of the SEIDF-BRA complex on the structural and physicochemical properties of rice starch was investigated. The mechanism of SEIDF and BRA interaction was studied through adsorption kinetic models, Fourier transform infrared spectroscopy (FT–IR), X-ray photoelectron spectroscopy (XPS) and thermogravimetric analysis (TGA). Furthermore, the effect of different ratios of the SEIDF-BRA complex on the pasting property, micromorphology, rheological behavior and thermal stability of rice starch was investigated, and potential interactive mechanism between SEIDF-BRA complex and rice starch were speculated. This study not only promotes the efficient utilization of shiitake mushroom stems but also contributes to the potential application of anthocyanin-DF complex in functional foods.

## 2 Materials and methods

### 2.1 Materials and chemicals

Shiitake mushroom stems were purchased from Nanyang, Henan. Black rice anthocyanins were purchased from Shingling Biotechnology Co., Ltd. (Xi'an, Shaanxi). Rice starch was purchased from Shanghai Yuanye Biotechnology Co., Ltd. (Shanghai, China). Fluorescein isothiocyanate (FITC) and fluorescent whitening agent 28 were supplied by Shanghai Yien Chemical Technology Co., Ltd. (Shanghai, China).

### 2.2 Preparation and modification of SEIDF

The shiitake mushroom stems were rinsed and impurities were removed. Next, the cleaned stems were dried at 60 °C, ground and sieved through an 80 mesh sieve. Then the resulting shiitake mushroom stem powder was added to distilled water (1:30, g/mL), and the pH value was adjusted to 8. Afterwards, 4% alkaline protease was added to the sample based on the weight of the shiitake mushroom stems powder, and the mixture was incubated in a 60 °C water bath for 30 min. After centrifugation, the pellet was freeze-dried to obtain shiitake mushroom stem insoluble dietary fiber (SMIDF).

The SE pretreatment of SMIDF was performed with a ECNKO steam explosion analysis test bed (ECN B.V. Netherlands). 200 g of SEIDF powder was weighed and placed in a stainless-steel reactor. The steam explosion pressure was set to 0.8 MPa and the time to 90 s for steam explosion treatment. The steam-exploded sample was then freeze-dried, ground into powder and sieved through an 80 mesh sieve to obtain the SEIDF.

### 2.3 Structural characterization of IDF

1 g of SMIDF and SEIDF samples were pressed into thin uniform sheets using a tablet press. Then, they were placed on a mica sheet and examined using a Bruker Dimension Fast scan atomic force microscope (Bruker Nano Surfaces Division, Germany). The prepared samples were scanned using a multifunctional scanning probe with a scanning frequency of 0.992 Hz. Image analysis was performed using Nano Scope Analysis.

### 2.4 Adsorption of black rice anthocyanins by SEIDF

1 g of SEIDF powder was mixed with 100 mL of 7 mg/mL BRA solution with continuous stirring for 60 min. After the adsorption process was completed, the suspension was centrifuged and the resulting supernatant was for the determination of the residual BRA concentration using a UV–Vis spectrophotometer at 520 nm based on a pre-established calibration curve. The adsorption capacity of SEIDF for BRA was determined following eq. (1). The precipitate collected from centrifugation was freeze-dried, obtaining the SEIDF-BRA complex.

(1) $$\mathrm{BRA}\;\text{adsorption capacity}\;\left(\mathrm{mg}/g\right)=\left(C_{0}-C_{t}\right)\times V/W$$

where *C*_0_ and *C*_*t*_ represent the first and equilibrium concentration of BRA solution (mg/mL), *V* represents the solution volume (mL), and *W* represents the weight of SEIDF absorbent (g).

Batch experiments were conducted to investigate the effects of different concentrations, pH values and temperatures on the adsorption capacity of SEIDF for BRA. The BRA solution concentrations were 1, 3, 5, 7, 9 and 11 mg/mL, the pH values were 3, 4, 5, 6, 7 and 8, the adsorption temperatures were 20, 25, 30, 35, 40 and 45 °C.

### 2.5 Preparation of SEIDF-BRA complex and SEIDF-BRA mixture

SEIDF-BRA complex was prepared under the optimal adsorption conditions. 1 g of SEIDF powder sample was mixed with 7 mg/mL BRA solution and the pH of the suspension was adjusted to 5. The suspension was shaken for 60 min in a temperature shaker (35 °C). Subsequently, the suspension was centrifuged and the precipitate was collected, which was named as SEIDF-BRA complex.

To clarify the interaction mechanism between SEIDF and BRA, the comparative mixture of SEIDF and BRA (SEIDF-BRA mixture) was prepared. Briefly, 35.3 mg of BRA and 1 g of SEIDF were weighed and thoroughly mixed using a vortex mixer to obtain the SEIDF-BRA mixture. The ratio of BRA to SEIDF in the SEIDF-BRA mixture was selected based on the adsorption capacity of SEIDF for BRA (35.3 mg/g) under optimal adsorption conditions (pH 5, 35 °C and BRA solution concentration of 7 mg/mL).

### 2.6 Adsorption kinetics study

According to Liu et al. (2023). The SEIDF was added into 7 mg/mL BRA solution according to Section 2.4 and the adsorption experiments were conducted. The adsorption time was set to 20, 40, 60, 80 and 100 min. After adsorption, the supernatant was separated by centrifugation and the residual anthocyanin levels at different time points were determined. The kinetic data of BRA adsorption by SEIDF were analyzed using pseudo-first-order and pseudo-second-order kinetic models (Chen et al., 2023).

### 2.7 Adsorption isotherm experiment

According to Wang et al. (2025), 1 g of SEIDF sample was added into 100 mL BRA solution with different concentrations, and the suspension was shaken for 60 min at the temperature of 35 °C. Freundlich and Langmuir isotherm models were used to describe the adsorption isotherm of BRA onto SEIDF.

### 2.8 Structural property analysis of SEIDF-BRA complex

#### 2.8.1 Fluorescence microscopy analysis

The sample morphology was performed using a fluorescence microscope (THUNDER Imager Tissue, shanghai, China) under three different light sources (visible light, green fluorescence and blue fluorescence). A DM suppression filter was used for the blue fluorescence excitation (355–389 nm) and green fluorescence excitation (465–495 nm) respectively. The samples were observed with a × 10 eyepieces and a × 20 objective lens.

#### 2.8.2 FT–IR analysis

A Fourier transform infrared spectrometer (Spectrum 100, PerkinElmer, UK) was employed to record the FT–IR spectra of the samples. 3 mg of samples were mixed and ground with an appropriate amount of KBr powder. The mixture was then pressed into a pellet, and infrared spectra were obtained over 500–4000 cm^−1^. The measurement was repeated for three times. Key metrics included the total crystallinity index (TCI), computed as the absorbance ratio at 1347 cm^−1^ versus 2917 cm^−1^, and the hydrogen bond intensity (HBI), derived from peak heights at 3401 cm^−1^ relative to 1347 cm^−1^ (Kong et al., 2026). In addition, the normalized intensities of hydroxyl and C—O peaks were calculated by the absorbance ratio at 3401 cm^−1^ and 1027 cm^−1^ relative to 2917 cm^−1^ respectively according to previous study (Liu et al., 2026).

#### 2.8.3 X-ray photoelectron spectroscopy (XPS)

5 mg of samples were weighed and placed into an X-ray photoelectron spectrometer (Thermon Fisher Scientific K-Alpha+, Shimadzu Corporation) for analysis. The test parameters were set as follows: chamber pressure 2.67 × 10^−7^ Pa, incident angle 45° and anode power 150 W.

#### 2.8.4 Thermal stability analysis (TGA)

A thermogravimetric analyzer (Netsch TG209F3, Germany) was employed to analyze the thermal stability of samples. The samples (3 mg) were placed in a crucible, sealed and heated from 30 °C to 500 °C. The heating rate was at 10 °C/min.

### 2.9 Effect of SEIDF-BRA complex on the properties of RS gel

#### 2.9.1 Preparation of composite gel and their RVA analysis

A Rapid Viscosity Analyzer (RVA 4500, Botong Ruhika Scientific Instrument, China) was used to measure the pasting property of samples. 5 g of RS was mixed with 0, 2%, 4%, 6%, and 8% SEIDF-BRA complex (*w*/w based on RS weight). Then the mixture was added with deionized water respectively to a final concentration of 15% and then transformed to an RVA aluminum container. The slurry was heated according to the test procedure. Parameters include pasting temperature (PT), breakdown viscosity (BV), setback viscosity (SV), peak viscosity (PV) and trough viscosity (TV) were recorded. The starch paste generated by the RVA was cooled and the resulting gel was designated according to the addition ratio of SEIDF-BRA complex: RS, SEIDF-BRA complex-2, SEIDF-BRA complex-4, SEIDF-BRA complex-6, SEIDF-BRA complex-8. A control sample was prepared using the same protocol but with the addition of 4% SEIDF (based on RS weight), and the control sample was named SEIDF-4. The selection of these additions was based on preliminary trials, which indicated that addition of SEIDF above 4% alone adversely affected RS gel property.

#### 2.9.2 DSC determination

The thermodynamic properties of the samples were analyzed using differential scanning calorimetry (DSC 250, TA Instruments, DE, USA). 3 mg of the sample was weighed into a crucible, and distilled water was added at a 1:3 (m/v) ratio. The crucible was sealed and equilibrated at 4 °C for 24 h, with an empty crucible used as a blank control. The temperature was increased from 25 °C to 110 °C and the heating rate was 10 °C/min.

#### 2.9.3 Texture property determination

The starch pastes generated by the RVA were equilibrated at 4 °C for 24 h. A texture analyzer (TA–XT plus, Stable Micro Systems, England) was employed to determine their texture properties. The probe was P/50, the test and return speeds were 1 mm/s, the compression ratio was 50% and the trigger pressure was 5 g.

#### 2.9.4 Rheological property determination

##### 2.9.4.1 Static rheological analysis

Static rheological measurements were performed using a MCR102 rheometer (Anton Paar GmbH, Austria) with a parallel plate of 50 mm diameter and at a 1 mm gap distance. The starch paste generated by the RVA was then loaded onto the parallel plate at 25 °C. Static rheological measurements were conducted with shear rate increasing from 0.1 to 100 s^−1^ and the data were fitted to a power-law model (Pourfarzad et al., 2021).

##### 2.9.4.2 Frequency scan analysis

The frequency sweep tests were performed at 1% strain amplitude. The frequency ranged from 0.1 to 10 Hz. G′, G″, and tan *δ* of the samples were determined.

#### 2.9.5 Effect of SEIDF-BRA complex on the microstructure of RS gel

##### 2.9.5.1 SEM analysis

The gel samples were generated according to Section 2.9.1 and then were freeze-dried. A scanning electron microscopy (S–3400 N, Hitachi Ltd., Japan) was employed to observe the cross-sectional morphology of samples. The sample was fixed on a metal post and sputter-coated with gold. SEM analysis was conducted at a voltage of 15 kV and a magnification of × 200.

##### 2.9.5.2 Observation using laser confocal microscopy (CLSM)

A confocal microscope (Zeiss LSM900, Germany) was employed to visualize the microstructure of the samples. Sample preparation was carried out as described in Section 2.9.1. 10 mg of gel was spread evenly on a glass slide. 250 μL of FITC (0.1%, w/v) and fluorescent whitening agent 28 (0.2%, w/v) were added to the sample and the sample was stained at room temperature for 30 min. The excitation wavelength was set to 488 nm.

#### 2.9.6 Effect of SEIDF-BRA complex on the crystalline structure of RS gel

##### 2.9.6.1 X-ray diffraction (XRD) analysis

The crystalline structure of the gel samples was determined using an XRD diffractometer (Arl Equinox 100, Thermo Fisher, America) according to Liang et al. (2024). To obtain retrograded starch samples, the starch pastes generated by the RVA were equilibrated at 4 °C for 7 days and then freeze-dried. Data were acquired in continuous mode at a diffraction angle (2θ) scanning rang of 5°–40° with an accelerating voltage of 40 kV and a current of 30 mA. The relative crystallinity of the samples was calculated using Jade 9.0 software (MDI Corporation, California, USA).

##### 2.9.6.2 Short-range ordered structure analysis

The short-range ordered structure of composite starch gel was characterized by FT–IR spectroscopy. The gel samples were generated according to Section 2.9.1 and then were freeze-dried. The FT–IR spectra were recorded on an FT–IR spectrometer from 4000 to 400 cm^−1^.The absorbance ratio of the bands at 1047 cm^−1^ to 1022 cm^−1^ (R_1047/1022_) was calculated after deconvolution using OMNIC 8.0 software.

#### 2.9.7 Sensory evaluation of composite gel

Sensory evaluation of composite gels was conducted according to Wu, Jia, et al. (2026) and Wu, Yi, et al. (2026) with slight modifications. Evaluations were carried out in the sensory laboratory of the College of Food and Bioengineering at Jilin Agricultural University. A panel of 20 trained assessors (10 male and 10 female, aged 22–30 years) participated in the sensory analysis. The starch gels were prepared according to Section 2.9.1 and stored at 4 °C for 7 days. The sensory attributes of the starch gel, such as appearance, aroma, taste, texture, mouthfeel, overall acceptability were assessed using a nine-point hedonic scale (1 = dislike extremely, 5 = neither like nor dislike, 9 = like extremely). The average score for each attribute was used for data analysis.

### 2.10 Statistical analysis

Each set of experiments was repeated independently three times and data were expressed as mean ± standard deviation. Statistical analyses were performed using IBM SPSS Statistics (SPSS Inc., Chicago, IL, USA) and significant differences were indicated by *p* < 0.05. Figures and tables were generated using Origin 2024 (Origin Lab, Northampton, MA, USA).

## 3 Results and analysis

### 3.1 Characterization of the IDFs

**Fig. 1:**
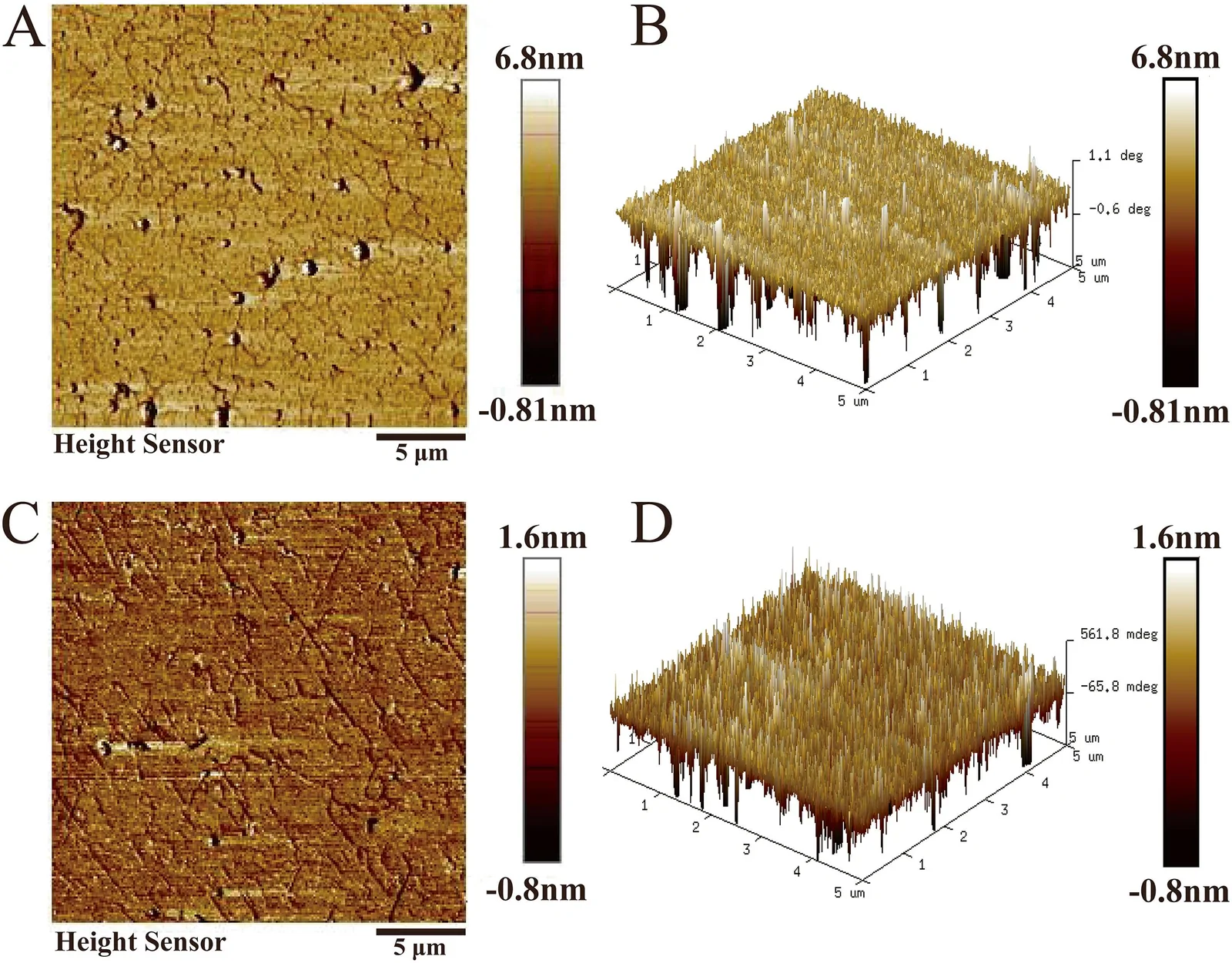
Atomic force microscopy scanning results of SMIDF and SEIDF: (A) and (C) are 2D diagrams of SMIDF and SEIDF respectively. (B) and (D) are 3D diagrams of SMIDF and SEIDF respectively.

Atomic force microscopy (AFM) can characterize the microscopic morphology of a sample through the interaction force between the probe and the sample (Gao et al., 2025). The 2D and 3D AFM images of SMIDF and SEIDF were shown in Fig. 1. Fig. 1A and C showed that SMIDF exhibited a denser and smoother surface, while after the steam explosion treatment the SEIDF surface became loosened and swelling. In addition, from the 3D image it can be seen that SMIDF exhibited loosely distributed low-profile protrusions. In contrast, SEIDF displayed more and significantly taller protrusions (Fig. 1B and D). Table 1 also showed that the average roughness (R_a_) and root mean square roughness (R_q_) of SEIDF were higher than those of SMIDF. The above results indicated that the structure of SEIDF became rough and porous with numerous folds after the steam explosion treatment. This structural change could lead to the exposure of the internal structure and provide more physical adsorption sites, thereby enhancing the binding capacity for BRA.

**Table 1:** Surface morphological parameters of fiber determined by AFM.

Sample	R_a_ (nm)	R_q_ (nm)
SMIDF	0.58 ± 0.01^b^	1.04 ± 0.02^b^
SEIDF	0.84 ± 0.01^a^	2.82 ± 0.03^a^

Note: R_a_ and R_q_ represent the average roughness and root mean square roughness of the fiber surface, respectively. Means (*n* = 3) in columns with different superscripts indicate significant differences (*p* < 0.05).

### 3.2 Optimization of adsorption condition

The effect of different adsorption pH, adsorption temperature and BRA solution concentration on the BRA adsorption capacity was shown in Fig. S1. The optimal conditions were pH of 5, adsorption temperature of 35 °C and BRA solution concentration of 7 mg/mL, which resulted in 35.3 ± 0.32 mg/g of adsorption capacity.

### 3.3 Adsorption kinetics

**Fig. 2:**
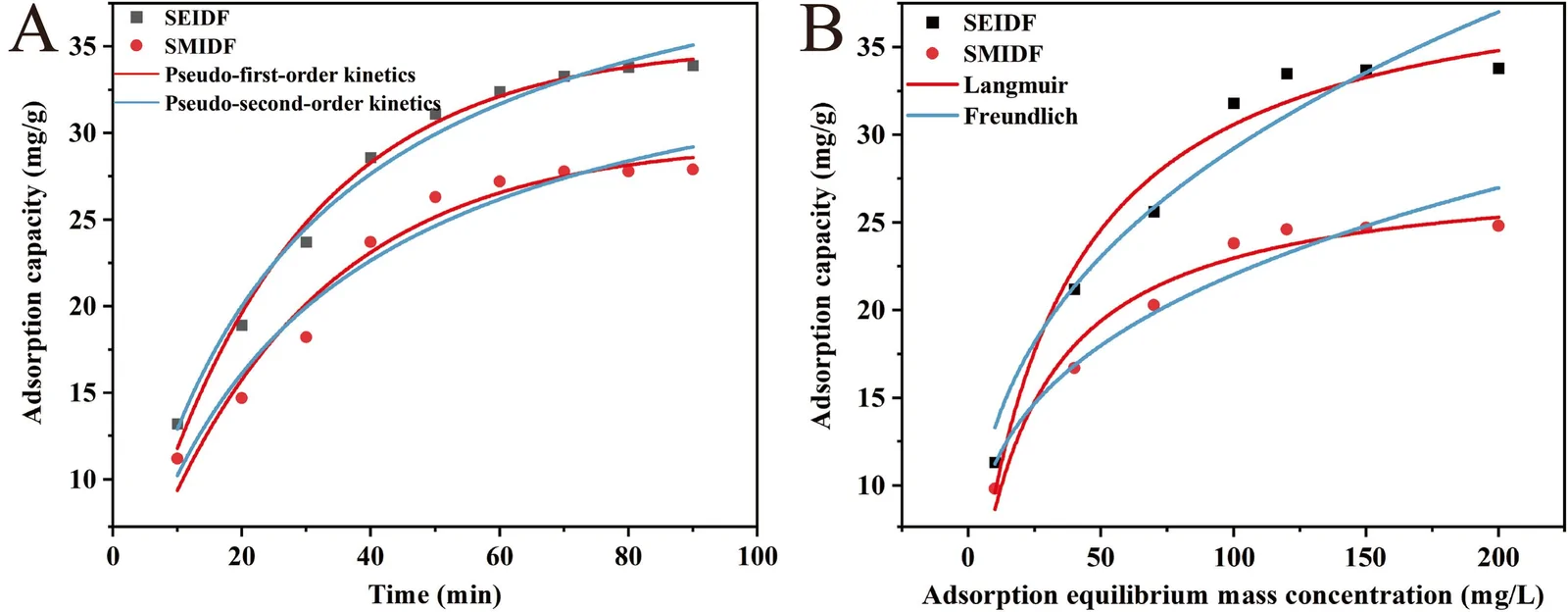
The adsorption curves of SMIDF and SEIDF: (A) represents the adsorption kinetic curves for SEIDF and SMIDF. (B) represents the adsorption isotherms of SEIDF and SMIDF.

Both the pseudo-first order and pseudo-second-order kinetic models were applied to the adsorption processes of SEIDF and SMIDF, as depicted in Fig. 2A. The BRA adsorption capacity of SEIDF and SMIDF were increased rapidly in the initial stage, followed by a gradual slowdown at approximately 60 min. The corresponding kinetic parameters were summarized in Table 2. The pseudo-first-order kinetic model for SMIDF and SEIDF showed higher regression coefficients (*R*^2^ = 0.967, 0.990). This suggested that the pseudo-first-order model was more appropriate for describing the adsorption kinetics. It was reported that the pseudo-first-order model is based on the assumption of a physical adsorption process, while the pseudo-second-order model typically implied chemisorption where valence forces were involved (Baba et al., 2026). Consequently, the adsorption of BRA onto SMIDF and SEIDF was primarily driven by physical interactions. The calculated equilibrium adsorption amounts (Q_e_ values) of SEIDF and SMIDF, which were deduced from the pseudo-first-order kinetic model, were 35.16 mg/g and 29.56 mg/g respectively. These two values were closer to the experimentally observed Q_e_ values compared to those from pseudo-second order model, indicating that the adsorption experiment data for SMIDF and SEIDF were better fitted by the pseudo-first order. In addition, it can be seen that SEIDF exhibited superior adsorption performance for BRA. Results of AFM analysis showed that after steam explosion, the structure of SEIDF became rough and porous with numerous folds after the steam explosion treatment. This structural change could lead to the exposure of the internal structure and provide more physical adsorption sites, thereby enhancing the binding capacity for BRA. A similar result has also been reported in our previous study. Our previous study reported that the shiitake mushroom stem insoluble dietary fiber after steam explosion treatment (S-MSIDF) exhibited higher adsorption capability for strawberry flavor, probably because steam explosion disrupted the glycosidic bonds in hemicellulose and cellulose, exposing more hydroxyl groups (–OH) and internal hydrophobic regions (such as C—O and C=O). Thus the flavor molecules could bind onto S-MSIDF via non-covalent interactions (Liu et al., 2026).

**Table 2:** SMIDF and SEIDF adsorption model parameters.

Model	Adsorption constant	SMIDF	SEIDF
Quasi-first-order dynamic model	R^2^	0.97	0.99
	K_1_	0.038 ± 0.0002^b^	0.040 ± 0.0001^a^
	Q_e_	29.6 ± 0.2^b^	35.1 ± 0.1^a^
Quasi-second-order dynamic model	R^2^	0.96	0.99
	K_2_	0.0096 ± 0.0002^a^	0.0090 ± 0.0001^b^
	Q_e_	38.0 ± 0.3^b^	44.7 ± 0.2^a^
Langmuir Isotherm model	R^2^	0.93	0.94
	Q_m_	28.1 ± 0.2^b^	40.4 ± 0.3^a^
	K_L_	0.044 ± 0.003^a^	0.031 ± 0.004^b^
Freundlich Isotherm model	R^2^	0.96	0.97
	1/*n*	0.29 ± 0.01^b^	0.34 ± 0.01^a^
	K_F_	5.7 ± 0.1^b^	6.1 ± 0.1^a^

Note: Q_e_ (mg/g) is the amount of BRA adsorbed at equilibrium, K_1_ is the pseudo-first-order rate constant, K_2_ is the pseudo-second-order rate constant. Q_m_ (mg/g) is the maximum adsorption capacity, K_L_ is the Langmuir adsorption constant, K_F_ is the Freundlich adsorption constant, 1/*n* is the adsorption intensity. Means (*n* = 3) in columns with different superscripts indicate significant differences (*p* < 0.05).

### 3.4 Adsorption isotherm

Adsorption isotherms depict how BRA distribute between the solution and the solid absorbent when it reached equilibrium. The isotherms of BRA adsorbed on SMIDF and SEIDF were shown in Fig. 2B. The fitting parameters and correlation coefficients of Freundlich and Langmuir isotherms were summarized in Table 2. The Freundlich constant 1/*n* reflects adsorption intensity, and the 1/*n* value were found to be 0.29 and 0.34, which indicated favorable adsorption of BRA onto the adsorbents (0.1 < *n* < 0.5). Additionally, the 1/*n* value of SMIDF was lower than that of SEIDF, suggesting that SEIDF had higher adsorption intensity for BRA than SMIDF.

The Freundlich model exhibited higher correlation coefficients (*R*^2^ = 0.9699, 0.9678) compared with the Langmuir model (*R*^2^ = 0.9375, 0.9384), indicating that the equilibrium adsorption process of BRA onto SEIDF and SMIDF was better described by the Freundlich model. The Freundlich isotherm model is typically recognized to represent multilayer adsorption dominated by physical adsorption. These findings confirmed that BRA was adsorbed onto SEIDF and SMIDF in a multilayer, heterogeneous manner (Wu et al., 2021). This mechanism was analogous to the adsorption behavior of cellulose for catechin as reported by Liu et al. (2018).

### 3.5 Investigation into the structure and properties of the SEIDF-BRA complex

#### 3.5.1 Fluorescence microscopy images of SEIDF-BRA complex

**Fig. 3:**
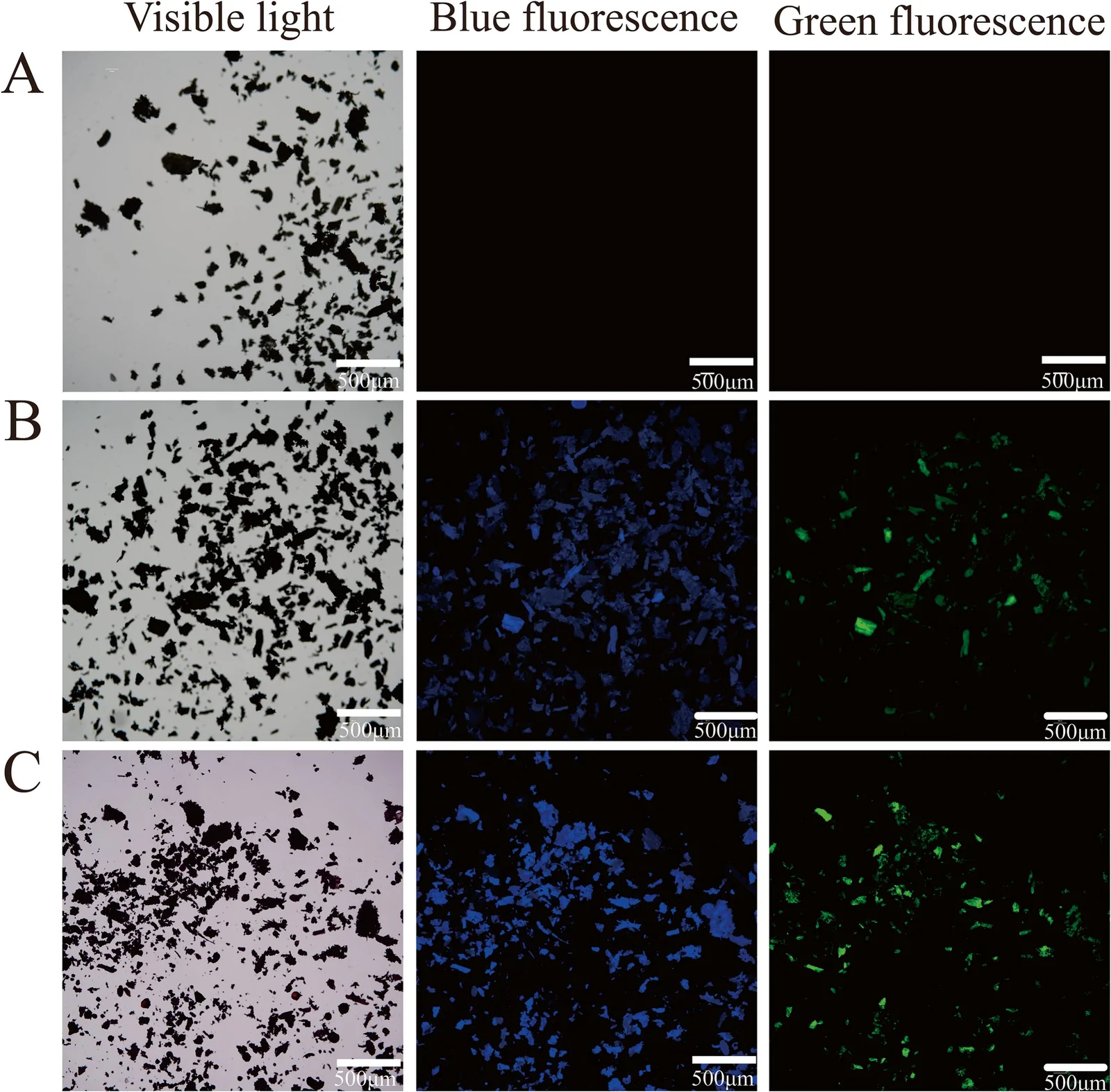
Fluorescence micrographs of SEIDF, SEIDF-BRA complex and SEIDF-BRA mixture: (A), (B) and (C) correspond to SEIDF, SEIDF-BRA complex and SEIDF-BRA mixture, respectively.

Fluorescence microscopy enables more direct visualization of the color reaction of BRA within SEIDF. As shown in Fig. 3A, the surface of SEIDF exhibited a rough and porous structure under visible light. However, SEIDF did not show blue or green fluorescence under excitation during 355–389 nm or 465–495 nm. This might be due to the fact that DF itself does not possess fluorescent property, thus it could not be visualized under blue and green fluorescence but under visible light. Fig. 3B and C show that the SEIDF-BRA complex and SEIDF-BRA mixture exhibited more dark spots under visible light. In addition, many blue or green fluorescence spots were detected in SEIDF-BRA complex and SEIDF-BRA mixture under blue and green fluorescence. Since BRA as a polyphenol possesses intrinsic fluorescence property and could be visualized under blue and green fluorescence illumination, it can be seen that of BRA was combined on the surface of SEIDF-BRA complex and SEIDF-BRA mixture.

By comparing the fluorescence images of SEIDF-BRA complex and SEIDF-BRA mixture, it was observed that the SEIDF-BRA complex displayed a weaker fluorescence response. Since the BRA concentrations in the SEIDF-BRA complex and SEIDF-BRA mixture were the same (35.3 mg/g), the weaker fluorescence response of SEIDF-BRA complex might be due to the fact that BRA was enclosed in the network structure of SEIDF and bind to the functional groups in the matrix due to the adsorption of BRA by SEIDF. Therefore, the SEIDF-BRA complex did not show strong fluorescence spots under blue and green fluorescence. In addition, previous studies have proved that interaction between DF (such as pectin, algin, polygalacturonase acid and aylan) and polyphenol (such as ferulic, *p*-coumaric acids, brown sugar polyphenols) may lead to quenching of the polyphenol's intrinsic fluorescence (Salazar-Medina et al., 2024; Wang et al., 2026). The underlying mechanism might involve the weak non-covalent binding of polyphenol molecules to active sites of DF influence the microenvironment of conjugated benzene rings in the polyphenol, thereby resulting in emission spectra associated with decay (Salazar-Medina et al., 2024). Thus the weaker fluorescence response of the SEIDF-BRA complex might be due to fluorescence quenching caused by interaction between BRA and SEIDF. In contrast, in the SEIDF-BRA mixture BRA were simply mixed with SEIDF, thus BRA was dispersed on the surface of DF and did not have strong interaction with SEIDF, which resulted in weak fluorescence quenching effect and relatively strong fluorescence response (Xu et al., 2022). These phenomena are consistent with the study by Chen, Cai, et al. (2025), Chen, Liu, et al. (2025) and Chen, Wang, et al. (2025), who reported that a complex of shiitake mushroom stem DF and tea polyphenol exhibited decreased fluorescence intensity compared with that of physical mixture. The results indicated that the SEIDF-BRA complex was not a simple physical mixture but rather a complex formed through adsorption.

#### 3.5.2 FT–IR

Fig. 5A displayed the infrared spectra of SEIDF, SEIDF-BRA complex and SEIDF-BRA mixture. The absorption peak at around 3400 cm^−1^ signified the stretching vibration of hydroxyl groups (O—H) (Liu et al., 2026). The peak near 2917 cm^−1^ corresponded to the stretching vibration of C—H in sugar rings. The peak at around 1588 cm^−1^ was attributed to C=C vibration, which is a characteristic peak of the aromatic ring skeleton of BRA (Zhong et al., 2025). The absorption peak near 1347 cm^−1^ corresponded to the bending vibration of methyl group (—CH_3_) on the sugar rings of hemicellulose and cellulose (Kaur et al., 2025). The prominent peak at around 1027 cm^−1^ represented methoxy C—O vibration in BRA, hemicellulose and cellulose (Hu et al., 2023).

Table S1 showed the TCI and HBI values and normalized peak intensities for key peaks. The TCI values of the samples followed the order: SEIDF-BRA complex < SEIDF-BRA mixture < SEIDF, which suggested that SEIDF-BRA complex had lower degree of crystallinity and more amorphous domains compared with SEIDF and SEIDF-BRA mixture. This might be due to the interaction of BRA with SEIDF interfered the polymerization of SEIDF. This is consistent with the study by Chen, Cai, et al. (2025), Chen, Liu, et al. (2025) and Chen, Wang, et al. (2025), who reported that the crystallinity index of the complex of shiitake mushroom stem DF and tea polyphenol was significantly decreased. The normalized hydroxyl-peak intensities and HBI values of the samples were in the following order: SEIDF-BRA complex > SEIDF-BRA mixture > SEIDF, which indicated that the hydrogen bonding interaction between BRA and SEIDF in the SEIDF-BRA complex was stronger (Valecillos et al., 2022). Table S1 also showed that the normalized C—O peak intensity of SEIDF-BRA mixture increased from 1.11 to 1.15, while for SEIDF-BRA mixture this value increased from 1.11 to 1.20, indicating the exposure of more C—O groups in the SEIDF-BRA complex.

Compared with SEIDF, the C=C vibration peak of SEIDF-BRA mixture was shifted from 1591 cm^−1^ to 1593 cm^−1^, while the C=C vibration peak of SEIDF-BRA complex was shifted further to 1603 cm^−1^. Moreover, the C=C vibration peak of SEIDF-BRA complex was sharper and more intense compared with that of SEIDF-BRA mixture. These results indicated that in the SEIDF-BRA complex the hydrophobic interactions between the aromatic ring skeleton of BRA and SEIDF were stronger (Caponio et al., 2024). In summary, these finding suggested that after the adsorption of BRA, the interactions between BRA and SEIDF through non-covalent binding occurs and more C—O groups and more hydroxyl groups were exposed, which contribute to the formation of more stable complex (Li et al., 2020).

#### 3.5.3 XPS

**Fig. 4:**
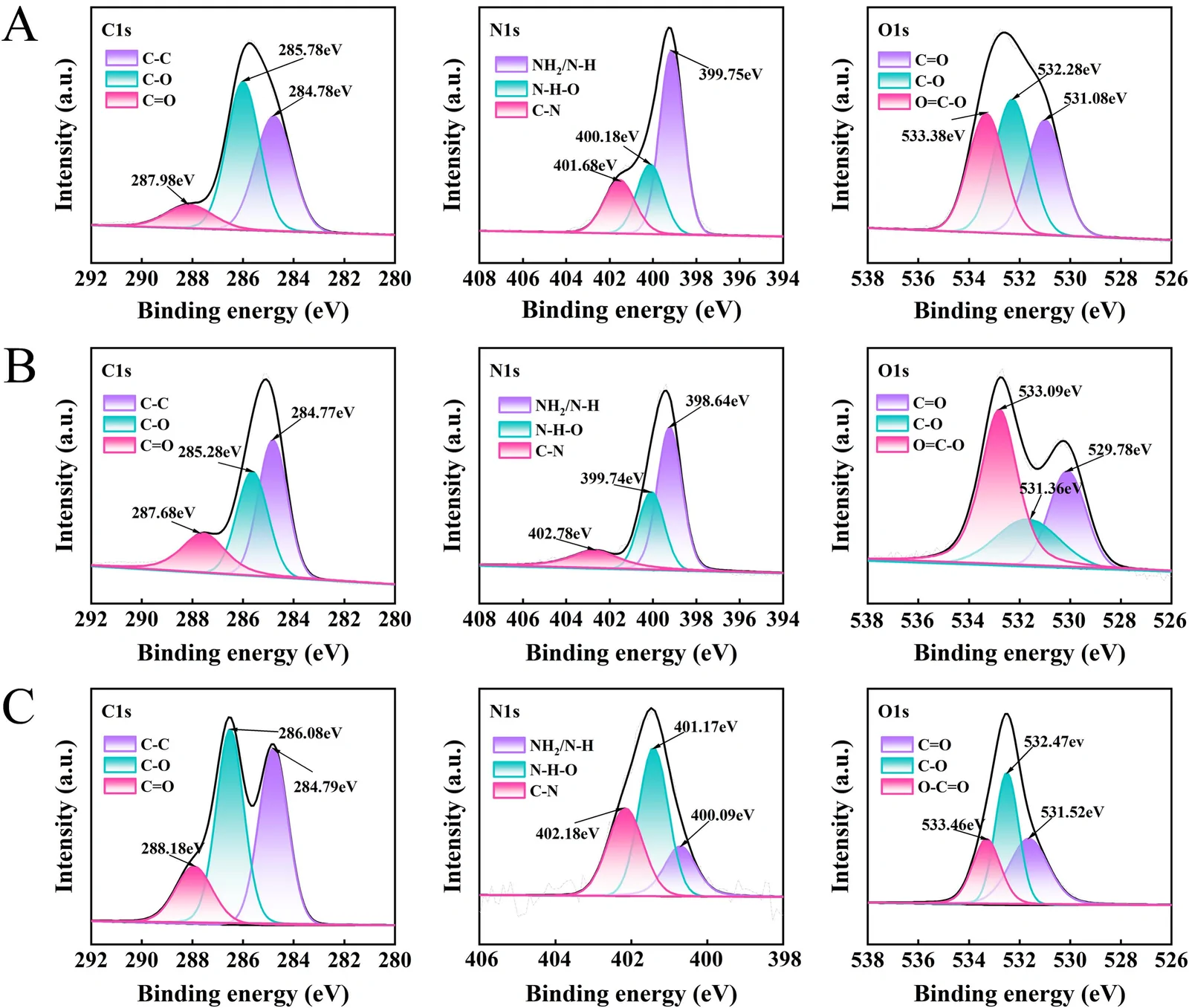
The XPS spectrum of SEIDF, SEIDF-BRA complex and SEIDF-BRA mixture: (A), (B) and (C) correspond to the Cls, Nls and Ols energy spectra of SEIDF, SEIDF-BRA complex and SEIDF-BRA mixture, respectively.

The XPS spectra of SEIDF, SEIDF-BRA complex, and SEIDF-BRA mixture were shown in Fig. 4. In the C1s spectrum of SEIDF, the three peaks at 287.98 eV, 285.78 eV, and 284.18 eV were attributed to C=O, C—O, and C—C bonds, respectively (Chen et al., 2021). After the adsorption of BRA, the binding energies of C=O and C—O peaks of the SEIDF-BRA complex decreased. This might be due to the fact that in the SEIDF-BRA complex the C=O and C—O groups participate in the formation of hydrogen bonds (C=O…H and C—O…H), leading to increased electron density on the carbons of C=O and C—O and decreased binding energy (Da Luz Corrêa et al., 2023). After the physical mixing of BRA and SEIDF, the binding energies of C=O and C—O peaks in the SEIDF-BRA mixture were increased, and the peak intensities of C=O and C—O in the SEIDF-BRA mixture were higher than those of SEIDF and SEIDF-BRA complex. This might be due to the increased contents of C=O and C—O groups after the addition of BRA in the SEIDF, and this result also indicated that in the C=O and C—O were not involved in the interactions between SEIDF and BRA in the mixture, which led to higher binding energies of C=O and C—O (Karkoosh et al., 2023).

In the N1s spectrum of SEIDF, three types of nitrogen were identified, namely NH_2_/N—H (398.98 eV), N—H—O (399.98 eV), and C—N (401.28 eV). It can be seen that after the adsorption of BRA, the binding energies of N—H—O and NH_2_/N—H were decreased in the SEIDF-BRA complex (Kou et al., 2024). It might be because the nitrogen atom for the N—H—O and NH_2_/N—H groups was a hydrogen bond donor, and thus it participated in the formation of hydrogen bonds during the formation of SEIDF-BRA complex, which lead to increased electron density on the nitrogen atom of N—H—O and NH_2_/N—H and decreased binding energy (Liu et al., 2025). However, after the physical mixing of BRA and SEIDF, the binding energies of C—N, N—H—O and NH_2_/N—H in the SEIDF-BRA mixture were all increased. This might be due to the increased concentrations of C—N, N—H—O and NH_2_/N—H groups after the mixing of BRA with SEIDF, and these groups did not involve in the interactions between SEIDF and BRA, which led to increased electron density on the nitrogen atom of C—N, N—H—O and NH_2_/N—H and thus higher binding energies (Yuan et al., 2025).

In the O1s spectrum of SEIDF, three peaks were also identified corresponding to C=O (531.18 eV), C—O (532.38 eV) and O=C—O (533.08 eV). It can be seen that after the adsorption of BRA, decrease in the binding energies of the C=O, C—O and O=C—O peaks were observed in the SEIDF-BRA complex, which might be due to the fact that these groups participated in the formation of hydrogen bonds during the formation of SEIDF-BRA complex (Wang et al., 2024). This result also is consistent with the analysis of C1s spectrum of SEIDF-BRA complex. In contrast, the binding energies of C=O, C—O and O=C—O peaks in the SEIDF-BRA mixture were increased, which might be due to the large concentrations of C=O, C—O and O=C—O groups after mixing with BRA and SEIDF, and that the C=O, C—O and O=C—O groups were not involved in the interactions between SEIDF and BRA in the SEIDF-BRA mixture (Xiao et al., 2020). These results are consistent with the FTIR analysis. FTIR analysis also showed that compared with SEIDF-BRA mixture, SEIDF-BRA complex exhibited a higher intensity of —OH group, indicating that hydrogen bonding interactions between SEIDF and BRA molecules in the SEIDF-BRA complex were stronger.

#### 3.5.4 Thermal stability analysis (TGA)

**Fig. 5:**
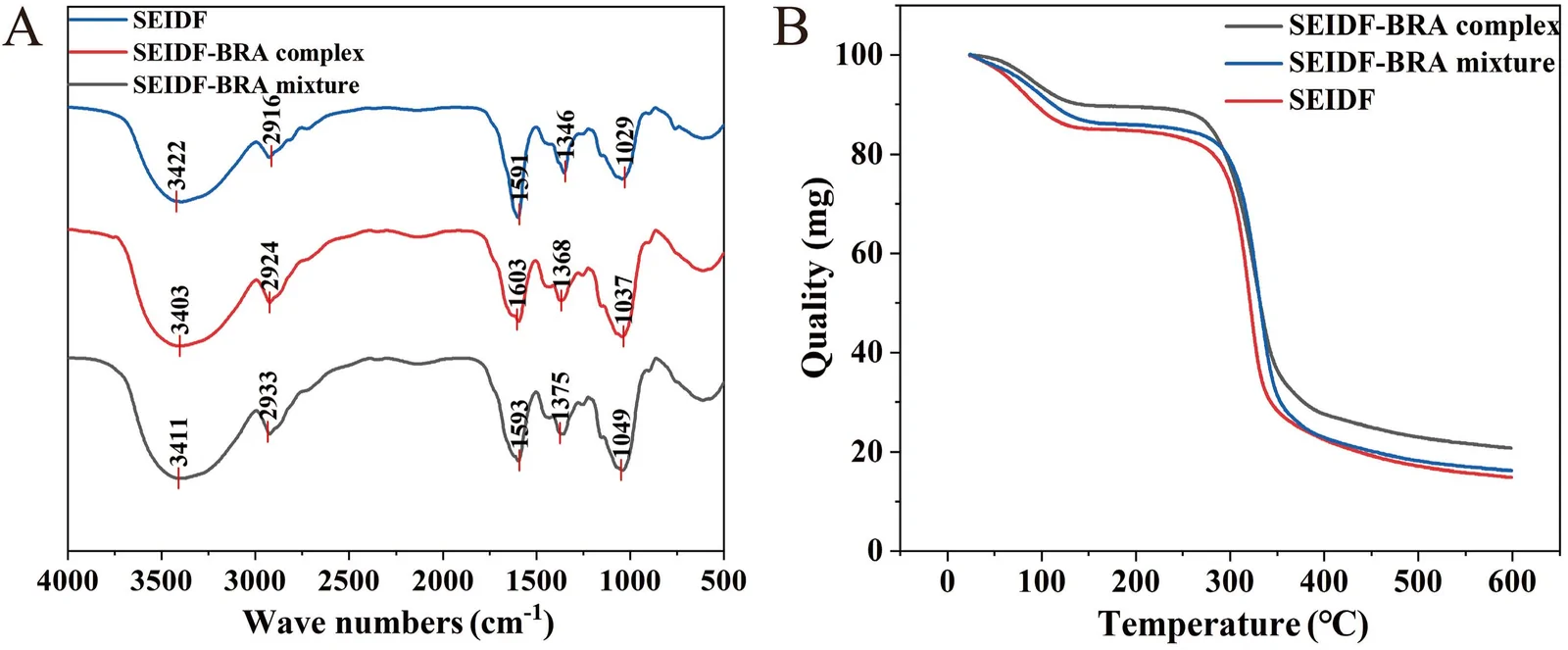
(A) represents the infrared spectra of SEIDF, SEIDF-BRA complex and SEIDF-BRA mixture. (B) represents the thermogravimetric curves of SEIDF, SEIDF-BRA complex and SEIDF-BRA mixture.

The TG curves of SEIDF-BRA complex, SEIDF-BRA mixture and SEIDF were shown in Fig. 5B. It can be seen that the pyrolysis process of the samples can be divided into three stages. In the first stage, all the three samples exhibited a slight mass loss within the temperature range of 60–150 °C, which was attributed to the volatilization of free or bound water or breakdown of hydrogen bonding (Li, Liu, et al., 2025; Li, Qin, et al., 2025). Compared to SEIDF-BRA mixture and SEIDF, the decomposition temperature of SEIDF-BRA complex at first stage shifted to higher temperature. This might be due to the hydrogen bonds between the SEIDF and BRA make the water in SEIDF-BRA complex require higher temperature to be removed. In addition, the SEIDF-BRA complex exhibited lower mass loss compared to the SEIDF-BRA mixture due to the strong interactions (such as hydrogen bonds) between the SEIDF and BRA in the SEIDF-BRA complex (Nawrocka et al., 2017). In the second stage, the samples display a large mass loss in the range of 250–350 °C, which might be due to degradation of cellulose and hemicellulose in SEIDF as well as BRA (Xin et al., 2026). The third stage was 350–600 °C, which was due to the carbonization of SEIDF and BRA. It can be observed that in the second and third stages of degradation, the SEIDF-BRA complex also exhibited lower mass loss compared to the SEIDF-BRA mixture. The result indicated that the adsorption of BRA make the SEIDF-BRA complex more stable because of the enhanced interactions such as hydrogen bond (Chen, Cai, et al., 2025; Chen, Liu, et al., 2025; Chen, Wang, et al., 2025). This finding is consistent with the study by Li et al. (2020), who reported that the complex of lotus root SDF and gallic acid has higher stability than the mixture of lotus root SDF and gallic acid.

### 3.6 Effect of SEIDF-BRA complex on the properties of RS

#### 3.6.1 Rapid viscosity analysis (RVA)

As shown in Table 3, compared with the pure RS sample, addition of SEIDF reduced the PV and TV values of the starch paste. Similar result was also reported by Liang et al. (2024), who found that the PV and TV of wheat starch were decreased as wheat bran IDF was added. This might be due to IDF adhering to starch granule surface minimized the inter-granule interactions, consequently resulting in lower PV and TV. However, the PV and TV values of starch paste with 2–8% SEIDF-BRA complex were higher than those of starch paste with SEIDF, and as the addition level of SEIDF-BRA complex increased, the PV and TV values of starch paste increased. This might be due to the fact that the adsorption of BRA onto SEIDF induces the SEIDF-BRA complex into a structure with more hydrogen bonds or other functional groups on the surface, Thus the SEIDF-BRA complex could interact with the leached-out amylose and amylopectin via intermolecular hydrogen bonding during gelatinization process, resulting in generation of a stable gel network and enhancement in the system's thickening effect (Wen et al., 2024).

**Table 3:** Effects of SEIDF-BRA complex on the gelatinization properties of RS gel.

Samples	PV (mPa·s)	TV (mPa·s)	BV (mPa·s)	SV (mPa·s)	PT (°C)
RS	2825 ± 4.08^d^	2045 ± 2.50^d^	874 ± 2.22^a^	794 ± 3.21^a^	87.73 ± 0.13^d^
SEIDF-4	2752 ± 3.26^f^	1996 ± 2.05^f^	792 ± 3.01^b^	786 ± 3.72^b^	85.32 ± 0.14^e^
SEIDF-BRA complex-2	2810 ± 4.28^e^	2024 ± 3.01^e^	786 ± 3.19^c^	745 ± 2.94^c^	88.01 ± 0.14^d^
SEIDF-BRA complex-4	2851 ± 4.39^c^	2050 ± 2.09^c^	775 ± 2.08^d^	734 ± 2.54^d^	89.81 ± 0.14^c^
SEIDF-BRA complex-6	2867 ± 5.05^b^	2104 ± 3.07^b^	761 ± 2.77^e^	724 ± 3.25^e^	90.36 ± 0.16^b^
SEIDF-BRA complex-8	2885 ± 4.06^a^	2116 ± 2.81^a^	751 ± 2.53^f^	715 ± 2.61^f^	90.74 ± 0.16^a^

Note: PV, TV, BV, SV, and PT represent the peak viscosity, through viscosity, breakdown viscosity, setback viscosity and pasting temperature. Means (*n* = 3) in columns with different superscripts indicate significant differences (*p* < 0.05).

The BV and SV values of starch paste with 2% SEIDF-BRA complex were lower than those of the RS and SEIDF-4 samples, and these two values decreased as the addition level of SEIDF-BRA complex increased. These results indicated that addition of SEIDF-BRA complex improved the thermal stability and anti-retrogradation properties of the gel system. This might be due to the non-covalent binding (such as hydrogen bond) between BRA and SEIDF inhibited the rearrangement of amylose, thus inhibiting starch short-term retrogradation (Luo et al., 2025).

PT is the temperature at which starch begins to expand and break down, reflecting the ease of starch gelatinization (Remya et al., 2018). It can be seen that the PT of starch paste decreased from 87.73 °C to 85.32 °C with the addition of SEIDF. A similar result was also reported by Liang et al. (2024), who found that the PT of wheat starch was decreased as wheat bran IDF was added. The possible mechanism is that the filling and dilution effect of IDF weakened the interactions between starch molecules, thus the structural constraint for starch swelling and rupture is reduced, consequently leading to the advancement of the pasting time and the lowering of PT (Zhuang et al., 2024). However, starch paste with 2–8% SEIDF-BRA complex exhibited higher PT than RS sample, which might be due to the fact that the non-covalent binding (such as hydrogen bond) between BRA and SEIDF promoted the intermolecular interactions between the SEIDF-BRA complex with the leached amylopectin and amylose, forming a protective coating layer and ultimately retarding starch gelatinization and increasing the PT of starch (Luo et al., 2025). Overall, the above results indicated that the addition level of SEIDF-BRA complex has a significant impact on the paste qualities of rice starch, and the non-covalent binding (such as hydrogen bond) between BRA and SEIDF could improve gelatinization viscosity, delay gelatinization process and inhibit the rearrangement of amylose during retrogradation.

#### 3.6.2 Differential scanning calorimetry analysis (DSC)

The thermal transition parameters of the samples measured by DSC were shown in Table 4. The starch gels with 2–8% SEIDF-BRA complex exhibited higher T_0_, T_p_ and T_c_ values compared with RS and SEIDF-4 samples, and with the increasing content of SEIDF-BRA complex, the T_0_, T_p_, and T_c_ values of the starch gel systems increased slightly. This indicated that SEIDF-BRA complex increased the pasting temperature of starch and enhanced the thermal stability of the starch granules, which is consistent with the findings from RVA analysis. In addition, the ∆H value of starch gel with 2–8% SEIDF-BRA complex was lower than that of SEIDF-4, and as the content of SEIDF-BRA complex decreased, the *∆*H value decreased. This might be because the non-covalent binding between BRA and SEIDF promoted the intermolecular interactions and physical entanglements between the SEIDF-BRA complex and the leached amylopectin and amylose, which interfered with the starch chain arrangement, prevented starch retrogradation and led to a lower *∆*H value (Lv et al., 2026).

**Table 4:** Effects of SEIDF-BRA complex on the thermodynamic properties of RS gel.

Samples	T_0_	T_P_	T_c_	*∆*H
RS	61.09 ± 0.18^e^	63.56 ± 0.18^d^	66.37 ± 0.12^e^	1.95 ± 0.02^b^
SEIDF-4	60.77 ± 0.19^e^	62.23 ± 0.22^e^	65.24 ± 0.13^f^	2.47 ± 0.02^a^
SEIDF-BRA complex-2	63.08 ± 0.19^d^	65.19 ± 0.20^c^	67.24 ± 0.11^d^	1.87 ± 0.01^c^
SEIDF-BRA complex-4	63.86 ± 0.18^c^	66.46 ± 0.19^b^	68.82 ± 0.14^c^	1.81 ± 0.02^d^
SEIDF-BRA complex-6	64.97 ± 0.19^b^	67.65 ± 0.21^a^	69.72 ± 0.12^b^	1.72 ± 0.01^e^
SEIDF-BRA complex-8	65.59 ± 0.19^a^	67.94 ± 0.23^a^	71.24 ± 0.15^a^	1.64 ± 0.02^f^

Note: T_0_ indicates onset temperature, T_p_ indicates peak temperature, T_c_ indicates conclusion temperature, *Δ*H indicates gelatinization enthalpy. Means (*n* = 3) in columns with different superscripts indicate significant differences (*p* < 0.05).

#### 3.6.3 Effect of SEIDF-BRA complex on the texture characteristic of RS

**Fig. 6:**
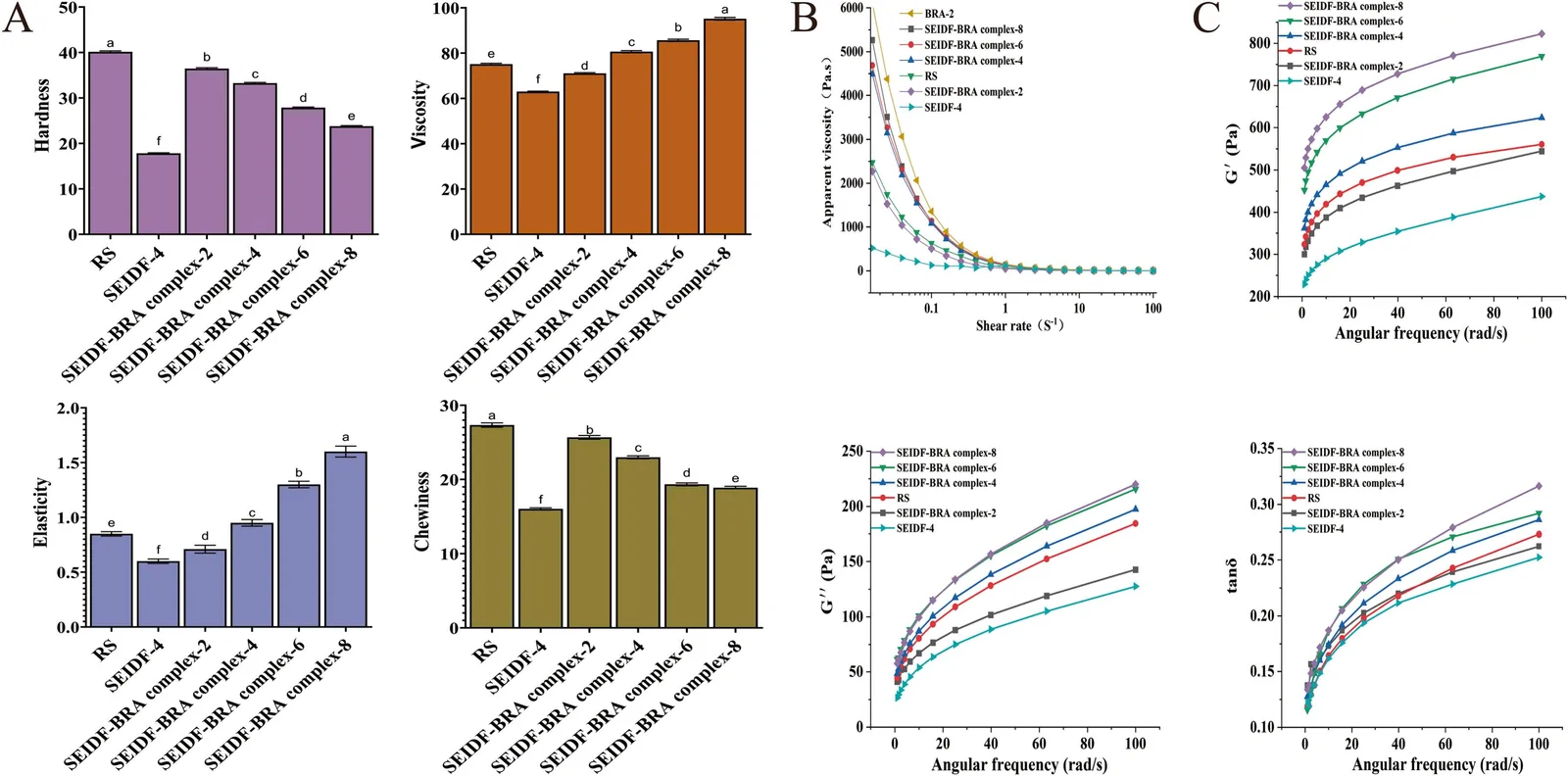
(A) represents the effect of SEIDF-BRA complex on the texture properties of RS gel. (B) represents the effect of SEIDF-BRA complex on the static rheological properties of RS gel. (C) represents the frequency dependence of the storage modulus (G′), loss modulus (G″) and loss tangent (tan *δ*) of RS gel with different levels of SEIDF-BRA complex.

It could be seen from Fig. 6A that the gels with 2–8% SEIDF-BRA complex exhibited lower hardness and chewiness and higher viscosity and elasticity compared with the RS sample. As the amount of the SEIDF-BRA complex increased, the viscosity and elasticity of the gel also increased. This might be due to the fact SEIDF-BRA complex could interact with starch through hydrogen bonding, which led to higher degree of cross-linking in the system. Thus, the stability, viscosity and elasticity of the gel network was improved. In addition, the interaction of SEIDF-BRA complex with starch impeded the orderly arrangement of straight-chain and branched starch during the retrogradation stage. For gels with 4% SEIDF, the hardness, chewiness, viscosity and elasticity were significantly decreased compared with those of RS sample. This might be because the physical entanglement and the competition of SEIDF for water hindered the interaction between starch molecules, which weakened the gel network (Nikinmaa et al., 2023).

#### 3.6.4 Effect of SEIDF-BRA complex on the rheological properties of RS

##### 3.6.4.1 Static rheology

Fig. 6B showed the static rheological curves of the samples. It can be observed that the shear-thinning behavior occurred for all the samples in the shear rate range of 0.1–100 s^−1^, as the apparent viscosity showed a dependence on shear rate (Dangi et al., 2020). The flow curves were fitted using the power-law model and the fitting parameters were shown in Table 5. The *R*^2^ values exceeded 0.95, indicating a good fit. The flow behavior index n of all samples was found to be less than 1 (0.58–0.66), which further confirmed the shear-thinning characteristics of the samples. The consistency coefficient K reflected the structural strength of the system (Li, Liu, et al., 2025; Li, Qin, et al., 2025). It can be seen that the K value of SEIDF-4 was significantly lower than that of RS sample. This might be due to the filling effect and the water competition behavior of IDF, which inhibited the interactions between starch molecules and leading to a loose structure. However, after the SEIDF-BRA complex was added, the K value was increased with the increase of the SEIDF-BRA complex addition ratio. This might be due to the interaction between SEIDF-BRA complex and the leached out amylose and amylopectin through intermolecular hydrogen bonding and physical entanglement, thereby the degree of internal cross-linking in the system was enhanced and the viscosity was improved (Guo et al., 2025). This is consistent with the previous results for viscosity in the RVA.

**Table 5:** Power-law model fitting parameters of the composite gel with different levels of SEIDF-BRA complex.

Samples	n	K (Pa.s)	R^2^
RS	0.63 ± 0.01^b^	283.38 ± 2.28^d^	0.96
SEIDF-4	0.58 ± 0.01^c^	131.20 ± 1.30^f^	0.96
SEIDF-BRA complex-2	0.60 ± 0.02^c^	144.85 ± 2.00^e^	0.95
SEIDF-BRA complex-4	0.64 ± 0.01^ab^	293.30 ± 1.08^c^	0.95
SEIDF-BRA complex-6	0.65 ± 0.01^ab^	325.06 ± 2.06^b^	0.99
SEIDF-BRA complex-8	0.66 ± 0.01^a^	331.58 ± 2.38^a^	0.97

Note: n is the fluidity index, and K is the sample consistency coefficient. Means (*n* = 3) in columns with different superscripts indicate significant differences (*p* < 0.05).

##### 3.6.4.2 Frequency scan

As shown in Fig. 6C, the storage modulus (G′) and loss modulus (G″) of all samples increased as the oscillatory frequency increased, and all samples displayed G′ exceeding G″, indicating that the gel samples was dominated by elasticity (Yin et al., 2025). The G′ and G″ values of SEIDF-4 sample were lower than those of RS sample, which might be due to the fact that the RS gel network was diluted by SEIDF and its viscoelasticity was weakened. However, for gels with SEIDF-BRA complex, the G′ and G″ values were increased as the addition level of SEIDF-BRA complex increased from 2% to 8%, and these values were higher than those of RS sample as the addition level of SEIDF-BRA complex were 4–8%, indicating that the elasticity and viscosity of the gel were effectively enhanced by the SEIDF-BRA complex.

The loss factor tan*δ* (tan*δ* = G″/G′) was an indicator of the relative contribution of the viscous and elastic components to the viscoelastic properties of starch gel (Fan et al., 2021). It was observed that the tanδ of all samples was less than 1, indicating that the samples were dominated by elasticity. As the addition amount of SEIDF-BRA complex increased from 2% to 8%, the tan*δ* of the samples gradually increased and exceeded that of RS and SEIDF-4 samples when the addition amount of SEIDF-BRA complex was higher than 6%, indicating that a higher level of SEIDF-BRA complex may led to a greater degree of viscous behavior of the starch gel. This might be due to the fact that the interaction between SEIDF-BRA complex and leached amylose limited the interactions of amylose molecules, which interfered with the orderly rearrangement of amylose and thus reduced the elasticity of the starch gel.

#### 3.6.5 Effect of SEIDF-BRA complex on the microstructure of RS (SEM and CLSM)

**Fig. 7:**
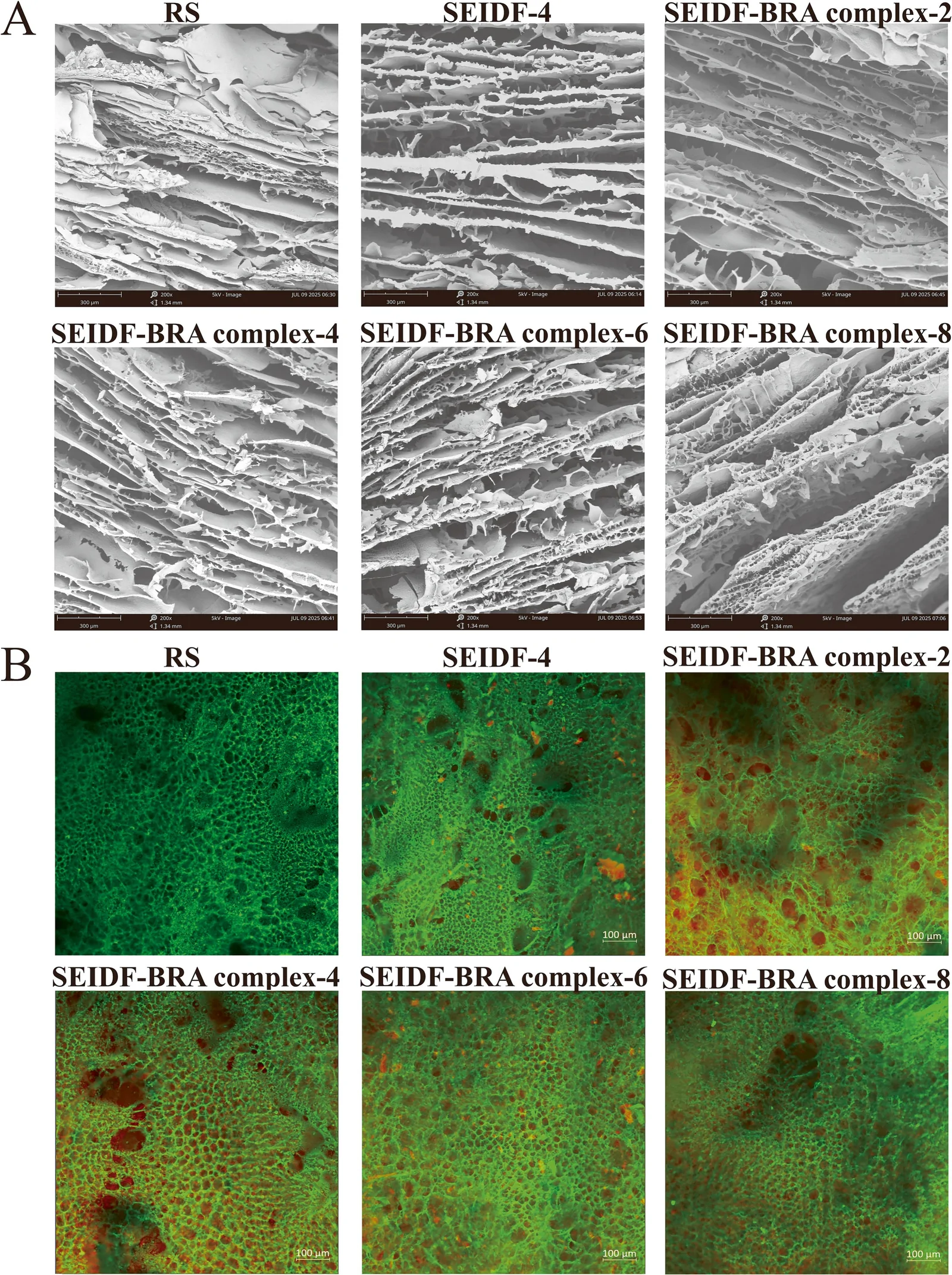
Scanning electron microscopy (A), laser confocal microscopy images (B) of RS gels with different levels of SEIDF-BRA complex.

The network structures of the gel samples were characterized by SEM. As shown in Fig. 7A, the RS gel exhibited a stacked lamellar structure with cross linking between some adjacent lamellar structures. With the introduction of SEIDF, there are more lamellar structures in the gel network and the cross-linking between the adjacent lamellar structures decreased. However, with the addition of SEIDF-BRA complex, the cross-linking between the adjacent lamellar structures increased. In particular, when the addition level of SEIDF-BRA complex was 8%, the starch gel formed a relatively regular network structure between the adjacent lamellar structures (Zhang et al., 2018). The SEIDF-BRA complex affected the lamellar structure and stack density of RS gel, and the cross-linking between the adjacent lamellar structures formed a porous network structure, which regulated the strength and viscosity of starch gels.

Further, the internal microstructure of gel samples was characterized by confocal laser microscopy. Starch was stained green with FITC and SEIDF was stained red with fluorescent whitening agent 28 (González-Quilen et al., 2020). As shown in Fig. 7B, compared with the RS sample, the SEIDF-4 sample presented a denser network structure and some larger SEIDF particles were interspersed within the starch gel network. For gel with 2–8% SEIDF-BRA complex, the porosity of the gel network increased, and the pores in the gels were more homogeneous than SEIDF-4 sample. In addition, the intensity of red fluorescence in the gel with 2–6% SEIDF-BRA complex was increased compared with that of SEIDF-4 sample, which might be due to the fact that SEIDF interacted with starch via hydrogen bonding and participated in the formation of the gel skeleton (Gu et al., 2026).

#### 3.6.6 Effect of SEIDF-BRA complex on the crystalline structure of RS

XRD is an effective method for characterizing the crystalline structure of starch. The diffraction patterns of the gel samples were shown in Fig. S2A. It can be observed that the RS sample exhibited a major diffraction peak at 20° after storage for 7 days, which was mainly due to the occurrence of starch retrogradation. Table S2 shows the relative crystallinity of the samples. It can be seen that the SEIDF addition significantly decreased the relative crystallinity of starch. However, compared to SEIDF, the SEIDF-BRA complex more obviously decreased the relative crystallinity of RS, and as the addition level of SEIDF-BRA complex increased, the relative crystallinity of RS gradually decreased. These results indicated that SEIDF-BRA complex is more effective at inhibiting amylose recrystallization. This might be due to the non-covalent binding (such as hydrogen bond) between BRA and SEIDF promoted the interactions of SEIDF-BRA complex with starch, which further inhibited the reorganization of the starch's crystalline structure and thereby reducing the relative crystallinity of RS. This aligns with the findings from RVA and DSC analyses.

To investigate the interactions between SEIDF-BRA complex and RS, as well as the structural changes of the composite gel, their infrared spectra were measured. As shown in Fig. S2B, all samples exhibited strong absorption peaks around 3400 cm^−1^, 2930 cm^−1^, 1650 cm^−1^ and 1020 cm^−1^, and compared to the control RS sample, no new characteristic peaks were observed in SEIDF-BRA complex addition groups or SEIDF addition groups, indicating that the addition of SEIDF-BRA complex did not alter the fundamental chemical structure of the starch, and the interactions between them were predominantly non-covalent (Wu, Jia, et al., 2026; Wu, Yi, et al., 2026). Fig. S2C shows the deconvolution spectra of samples from 1200 cm^−1^ to 800 cm^−1^. The absorption peaks at 1047 cm^−1^ and 1022 cm^−1^ represent the ordered structures in the crystalline region and the amorphous structures in the non-crystalline region of starch, respectively. A higher R_1047/1022_ value indicates a greater proportion of ordered structures within the starch molecules (Luo et al., 2025). As shown in Table S2, the R_1047/1022_ values of the starch paste with 2–8% SEIDF-BRA complex were significantly lower than that of the RS and SEIDF-4 samples, and the ratio decreased gradually as the SEIDF-BRA complex concentration increased, which indicates that SEIDF-BRA complex is more effective than SEIDF in reducing the short-range ordered structure of starch. This might be due to the SEIDF-BRA complex is more likely to integrate into the starch structure during retrogradation, increasing the distance between starch molecules and delaying the formation of hydrogen bonds (Zheng et al., 2025).

#### 3.6.7 Sensory properties of composite gel

The effect of SEIDF-BRA complex on the sensory properties of starch gel is shown in Fig. S3. It can be seen that on day 1 the aroma score of starch gel gradually increased with increasing addition levels of the SEIDF-BRA complex, and the aroma scores of starch gel containing 2–8% SEIDF-BRA complex were higher than those of RS and SEIDF-4 samples. This might be due to the non-covalent binding between BRA and SEIDF enhanced the retention capacity of SEIDF-BRA complex for rice flavor compounds. On day 1, starch gel with 4% SEIDF-BRA complex added received the highest scores for appearance, taste, texture, mouthfeel and overall acceptability. This is likely because the moderate addition imparted an appealing purple color to starch gel, while also improving the viscoelasticity and reducing the hardness of starch gel. In contrast, excessive addition resulted in a dark color, a slightly astringency taste, overly low hardness and a sticky, soft texture. After 7 days of storage, the starch gel containing 4% SEIDF-BRA complex exhibited higher scores in the appearance, aroma, taste, texture, mouthfeel and overall acceptability compared to the RS and SEIDF-4 samples. This might be due to the fact that moderate addition of SEIDF-BRA complex effectively suppressed the retrogradation and oxidative degradation of starch gels.

#### 3.6.8 Potential mechanism interpretation for the SEIDF-BRA complex formation and their influence on RS gel property

**Fig. 8:**
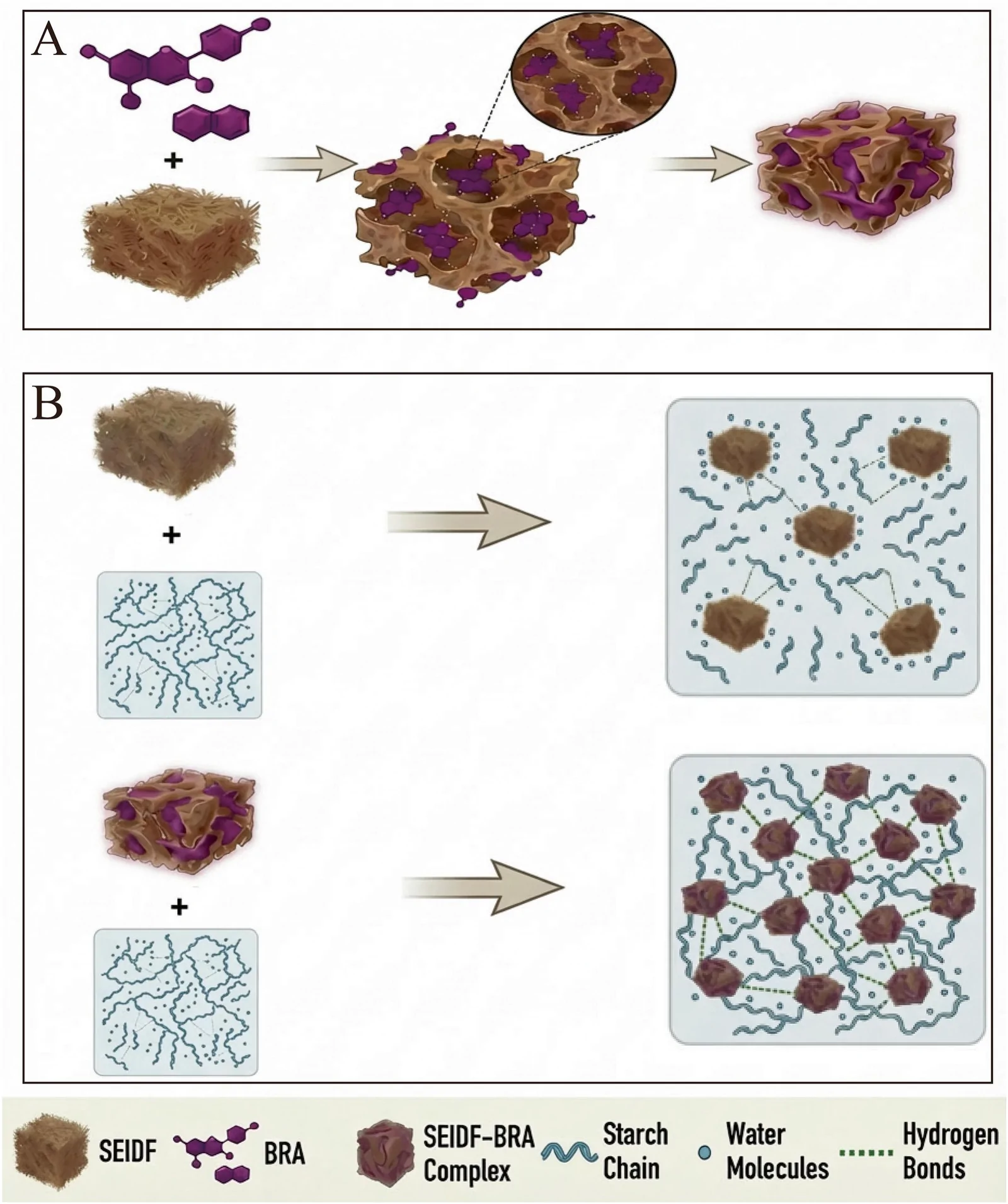
Schematic diagram of the formation of SEIDF-BRA complex (A), mechanism of the SEIDF-BRA complex effect on the RS gel structure (B).

As shown Fig. 8A, the structure of SEIDF became rough and porous with numerous folds after the steam explosion treatment. This structural change could lead to the exposure of the internal structure and provide more physical adsorption sites, thereby enhancing the binding capacity for BRA. After the adsorption of BRA onto SEIDE, BRA was encapsulated into the network structure of SEIDF to form a stable complex by interactions such as hydrogen bonding. The non-covalent binding (such as hydrogen bond) between BRA and SEIDF was stronger and more groups (such as C—O) were exposed in the SEIDF-BRA complex. The mechanism of SEIDF-BRA complex impact on the RS gel structure was shown in Fig. 8B. With the addition of SEIDF, the water competition behavior and filling effect of IDF may hinder the interactions between starch chains, reducing the cross-linking of gel network and thus reducing the viscosity and elasticity of gel. However, in SEIDF-BRA complex the non-covalent binding between BRA and SEIDF promoted the intermolecular interactions between the SEIDF-BRA complex with the leached amylopectin and amylose, which reduced the short-range ordered structure of starch, improve gelatinization viscosity, delay gelatinization process and inhibited the reorganization of the starch's crystalline structure. In addition, the nanoscale features of the SEIDF-BRA complex also exerted a significant influence on RS gelatinization. Our previous study demonstrated that after the adsorption of polyphenol onto SEIDF, the average diameter *D*_[3,2]_ of the complex increased, whereas its relative crystallinity decreased. The increase in average diameter *D*_[3,2]_ was attributed to the polyphenols entering the pores of dietary fiber, which promoted swelling of the SEIDF structure. The reduction in relative crystallinity likely resulted from strong interactions (such as hydrogen bonding) between SEIDF and polyphenols, which disrupted the crystalline structure of the dietary fiber, thereby promoting the swelling of the rigid fiber structures and increasing the available surface area (Chen, Cai, et al., 2025; Chen, Liu, et al., 2025; Chen, Wang, et al., 2025). Owing to its enlarged diameter and surface area, the SEIDF-BRA complex could encapsulate starch granules or fill the spaces between starch granules. Such physical entrapment is expected to retard starch gelatinization and increase gel viscosity.

## 4 Conclusion

In this study, a complex of SEIDF and BRA were constructed. The interactions between SEIDF and BRA, the structural characteristics of the SEIDF-BRA complex and the effect of the SEIDF-BRA complex on the starch gel were studied. AFM characterization confirmed that the steam explosion significantly enhanced the surface porosity and roughness of SEIDF, providing sufficient adsorption sites for BRA. Fluorescence microscopy revealed that SEIDF-BRA complex exhibited weaker fluorescence intensity, which might be due to the entrapment of BRA in the network structure of SEIDF or fluorescence quenching caused by interaction between BRA and SEIDF. Results of adsorption kinetics and isotherm, FT–IR and XPS analyses revealed that BRA was adsorbed onto SEIDF in a multilayer manner, and BRA bound to SEIDF via non-covalent interactions (such as hydrogen bonds). TGA analysis showed that SEIDF significantly improved the thermal stability of BRA. Furthermore, the SEIDF-BRA complex exerted a superior regulatory effect on RS compared with SEIDF alone. The results indicated that introducing SEIDF-BRA complex significantly increased the gelatinization temperature and thermal stability of RS, inhibited starch retrogradation, effectively improved viscoelasticity and texture property of starch gel. SEM and CLSM observations revealed that the SEIDF-BRA complex enhanced the porosity and cross-linking of the gel network. Furthermore, FT–IR and XRD analyses indicated that addition of SEIDF-BRA complex significantly decreased the relative crystallinity and short-range order degree of RS, which might be due to the non-covalent binding between BRA and SEIDF that promoted the SEIDF-BRA complex's binding affinity with starch. Sensory evaluation confirmed that moderate incorporation of the SEIDF-BRA complex not only enhanced the appearance, aroma, taste, texture, mouthfeel and overall acceptability, but also retarded the spoilage of starch gels. These findings implied that the SEIDF-BRA complex could be an interesting functional food ingredient, which could provide good compatibility and processing suitability to develop healthier starch-based foods. However, food is a highly complex system that comprises various essential components, such as starch, protein, lipids and other vital nutrients. Therefore, future work will focus on interactions of SEIDF-BRA complex with other food components (such as protein and lipids) and their effect on the textural attributes and functional properties of food. In addition, the structural evolution and bioavailability of SEIDF-BRA complex during in vitro digestion and during gut microbiota fermentation will be studied in our future work.

## CRediT authorship contribution statement

**Hongxiu Fan:** Writing – original draft, Methodology, Conceptualization. **He Gao:** Investigation, Data curation. **Yumeng Zhang:** Methodology, Formal analysis. **Meixuan Du:** Formal analysis, Data curation. **Tingting Liu:** Writing – review & editing, Data curation. **Dawei Wang:** Methodology, Investigation. **Hongcheng Liu:** Writing – review & editing, Funding acquisition. **Yanrong Zhang:** Writing – review & editing, Conceptualization.

## Declaration of competing interest

The authors declare that they have no known competing financial interests or personal relationships that could have appeared to influence the work reported in this paper.

## Data Availability

Data will be made available on request.
