# Supplementary material for: Preparation of modified shiitake mushroom stem insoluble dietary fiber and black rice anthocyanin complex and its effects on the gel properties, rheological properties and microstructure of rice starch
Source: Food Chem X. 2026 Aug 20;38:104344. doi: 10.1016/j.fochx.2026.104344 (PMC13524702; doi:10.1016/j.fochx.2026.104344)
Supplement: Supplementary file 1 [file mmc1.docx]

**Supplementary data**

**Fig. S1.** Effect of different pH value, adsorption temperature and BRA solution concentration on the BRA adsorption capacity.

**Fig. S2.** X-ray diffractograms (A) and FT–IR spectra (B: original spectra; C: deconvoluted spectra) of RS gels with different levels of SEIDF-BRA complex.

**Fig. S3.** Sensory profile of RS gels with different levels of SEIDF-BRA complex, measured on day 0 (A) and day 7 (B).

**Table S1.** The total crystallinity index (TCI), hydrogen bond intensity (HBI) and normalized key peak intensities of SEIDF-BRA complex and SEIDF-BRA mixture.

| Sample | TCI | HBI | Normalized hydroxyl peak intensity | Normalized C-O peak intensity |
| --- | --- | --- | --- | --- |
| SEIDF | 0.82 ± 0.02^a^ | 1.25 ± 0.025^c^ | 1.45 ± 0.03^c^ | 1.11 ± 0.015^c^ |
| SEIDF-BRA mixture | 0.76 ± 0.02^b^ | 1.58 ± 0.03^b^ | 1.72 ± 0.03^b^ | 1.15 ± 0.01^b^ |
| SEIDF-BRA complex | 0.68 ± 0.01^c^ | 1.92 ± 0.04^a^ | 2.05 ± 0.04^a^ | 1.20 ± 0.02^a^ |

Note: Means (*n* = 3) in columns with different superscripts indicate significant differences (*p* < 0.05).

**Table S2.** The short-range structural parameter and relative crystallinity of samples.

| Samples | Relative crystallinity (%) | R_1047/1022_ |
| --- | --- | --- |
| RS | 32.55 ± 0.82^a^ | 1.15 ± 0.01^a^ |
| SEIDF-4 | 29.76 ± 0.76^b^ | 1.14 ± 0.02^ab^ |
| SEIDF-BRA complex-2 | 27.93 ± 0.62^c^ | 1.12 ± 0.02^b^ |
| SEIDF-BRA complex-4 | 25.81 ± 0.56^d^ | 1.10 ± 0.02^c^ |
| SEIDF-BRA complex-6 | 23.10 ± 0.53^e^ | 1.08 ± 0.02^d^ |
| SEIDF-BRA complex-8 | 20.32 ± 0.45^f^ | 0.98 ± 0.01^e^ |

Note: Means (*n* = 3) in columns with different superscripts indicate significant differences (*p* < 0.05).
